# Insect tissue-specific vitellogenin facilitates transmission of plant virus

**DOI:** 10.1371/journal.ppat.1006909

**Published:** 2018-02-23

**Authors:** Yan Huo, Yuanling Yu, Liying Chen, Qiong Li, Mengting Zhang, Zhiyu Song, Xiaoying Chen, Rongxiang Fang, Lili Zhang

**Affiliations:** 1 State Key Laboratory of Plant Genomics, Institute of Microbiology, Chinese Academy of Sciences, Beijing, China; 2 National Plant Gene Research Center, Beijing, China; 3 University of the Chinese Academy of Sciences, Beijing, China; Fujian Agriculture and Forestry University, CHINA

## Abstract

Insect vitellogenin (Vg) has been considered to be synthesized in the fat body. Here, we found that abundant Vg protein is synthesized in *Laodelphax striatellus* hemocytes as well. We also determined that only the hemocyte-produced Vg binds to Rice stripe virus (RSV) *in vivo*. Examination of the subunit composition of *L*. *striatellus* Vg (LsVg) revealed that LsVg was processed differently after its expression in different tissues. The LsVg subunit able to bind to RSV exist stably only in hemocytes, while fat body-produced LsVg lacks the RSV-interacting subunit. Nymph and male *L*. *striatellus* individuals also synthesize Vg but only in hemocytes, and the proteins co-localize with RSV. We observed that knockdown of *LsVg* transcripts by RNA interference decreased the RSV titer in the hemolymph, and thus interfered with systemic virus infection. Our results reveal the sex-independent expression and tissue-specific processing of LsVg and also unprecedentedly connect the function of this protein in mediating virus transmission to its particular molecular forms existing in tissues previously known as non-Vg producing.

## Author summary

Rice stripe virus (RSV), which is completely dependent on *Laodelphax striatellus* for transmission between host plants, can also be vertically transmitted from the mother insect to its offsprings. Passing through the insect hemolymph is an essential step for both kinds of viral transmission. In this study, we found that RSV binds to *L*. *striatellus* vitellogenin (LsVg) in hemocytes. This tissue-specific LsVg-RSV interaction protects the virus for survival in the hemolymph and enhances both subsequent types of viral transport. More excitingly, we characterized novel and important properties of the insect form of Vg, an indispensable protein in almost all oviparous animals. In our study, we revealed for the first time that insect Vg transported into the ovary is also produced by tissues other than the fat body. Furthermore, we identified the tissue-specific molecular form of Vg responsible for its biological function in virus vertical transmission, uncovered non-female expression of Vg, and determined its function in virus horizontal transmission. These findings provide novel insights into plant-virus transmission within the vector, an important yet less explored part of the virus life cycle.

## Introduction

Rice stripe disease is a serious problem during rice production, with epidemics occurring repeatedly in China, Japan and Korea [1–3]. Transmission of the causative pathogen, Rice stripe virus (RSV), is completely dependent on insect vectors, the most important of which is the small brown planthopper (SBPH; *Laodelphax striatellus*) [4]. RSV is transmitted by *L*. *striatellus* in a persistent-propagative manner [4]. The RSV filamentous ribonucleoprotein particles (RNPs) are ingested by *L*. *striatellus* individuals feeding on RSV-infected plants. Once inside the insect, the virus invades the midgut epithelium to establish infection; it then spreads within the gut and disseminates into the hemolymph. From the hemolymph, the virus further infects various *L*. *striatellus* tissues, including the salivary glands. RSV is then horizontally transmitted from the salivary glands into a healthy plant, and also invades the female ovaries, from where it is vertically transmitted to the offspring [5, 6]. Vertical transmission results in naturally existing RSV-infected *L*. *striatellus*, which presents a further challenge in disease control.

RSV RNPs contain four single-stranded RNAs, and the major nucleocapsid protein (CP) encoded by the ORF at the 5’ half of the viral complementary RNA3 [4]. Thus, CP is considered the key viral component for specifically interacting with the vector components and plays roles in RSV transmission. Recently we demonstrated that CP interacts with *L*. *striatellus* vitellogenin (Vg) *in vitro*, and that the virions co-localize with Vg in the insect germarium [5]. RSV accomplishes its vertical transmission by binding to Vg and via the uptake of Vg by developing oocytes [5]. In the present study, we focused on the molecular events of the Vg–RSV interaction prior to oocyte penetration. Detailed knowledge of virus transmission mechanisms is required for the design of novel disease control strategies.

Vgs are precursors of the major egg storage proteins in many oviparous animals [7, 8]. Insect Vg is usually synthesized extra-ovarially by the fat body. After processing and modification, also in the fat body, the protein is secreted into the hemolymph and taken up by oocytes via receptor-mediated endocytosis [9–12]. All the known Vgs have been reported to possess modifications; however, the extent of Vg modifications varies considerably. Vg undergoes co- and post-translational modifications, as well as proteolytic cleavage [7, 12–14]. The primary Vg precursor separates into Vg subunits upon proteolytic cleavage. All insect Vgs, excluding those of the honeybee suborder *Apocrita*, are cleaved *in vivo* at the tetra-residue motif R-X-R/K-R by subtilisin-like endoproteases [8, 15–18]. This conserved R-X-R/K-R motif is located near the N-terminus and is flanked by polyserine tracts (see review [7]). Cleavage at this motif gives rise to two subunits, one large (140–190 kDa) and one small (40–60 kDa) (see review [19]). In some insects, the Vg precursor contains additional RXXR motifs, and the large subunit is further cleaved into two medium-sized (~90–110 kDa) polypeptides [17, 20–22]. Vg subunits produced by proteolytic cleavage are usually assembled in tissues and secreted into the hemolymph as oligomeric proteins. However, only the small subunit is secreted into the hemolymph, while the large subunit is consumed in the fat body [23].

Vg was initially regarded as a female-specific protein; however, Vg synthesis, albeit in small quantity, has been shown to occur in males and sexually immature animals, indicating that the function of Vg extends beyond serving as an energy reserve for the nourishment of developing embryos [14, 24, 25]. In recent years, accumulating data have shown that Vg from fish plays a role in immune responses [26–32], either as a pattern recognition molecule to recognize bacteria, or as an opsonin to enhance macrophage phagocytosis [27, 33]. Moreover, Vg has been reported to directly kill bacteria via interaction with lipopolysaccharides and lipoteichoic acid present in bacterial cell walls [28], and to neutralize viruses by binding to and creating cross-links between virions [26]. A few studies on the immunological properties of Vg in species other than fish have also been reported. For example, Vg of the mosquito *Anopheles gambiae* Vg is able to interfere with the anti-plasmodium response by reducing the parasite-killing efficiency of the antiparasitic factor TEP1 [34]. Vg in honeybees also has immunological binding properties, and, moreover, is able to mediate trans-generational immunity by transporting microbe-derived molecules into developing eggs [35].

In a previous study, we have examined the interaction between *L*. *striatellus* Vg (LsVg) and the RSV CP and addressed the molecular interaction and its function in mediating RSV vertical transmission [5]. In the present investigation, we further explored molecular details of the RSV–Vg interaction occurring prior to ovary-uptake. We found that only the Vg produced by and processed in insect hemocytes can interact with RSV. This molecular form of Vg is also produced by non-female *L*. *striatellus*, in hemocytes only and facilitates RSV transmission.

## Results

### Multiple tissues of female *L. striatellus* expressed Vg

Our previous study indicated that RSV RNPs entered the *L*. *striatellus* oocyte by binding to the Vg protein before reaching the germarium [5]. To ascertain in which tissue(s) the RSV–LsVg interaction occurs, we analyzed the expression levels of *LsVg* in various female *L*. *striatellus* tissues and carried out experiments to localize the LsVg protein. Tissues analyzed included the fat body, hemocytes, the midgut and salivary glands, all of which have been proposed previously to be involved in the transmission of persistent propagative viruses.

**Fig 1:**
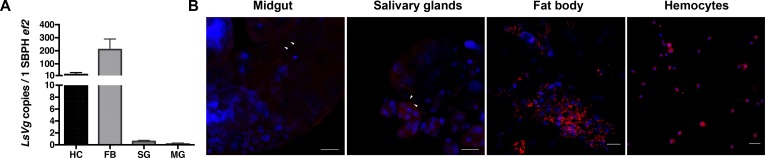
Gene expression and protein distribution of *Laodelphax striatellus* vitellogenin (LsVg) in tissues of the female insects. **A.** Distribution of *LsVg* mRNA in different tissues of female insects determined by qPCR. Both the mean and SD were calculated from three independent experiments, with four mRNA samples per experiment. Ef2, *L*. *striatellus* elongation factor 2 gene; HC, hemocyte; FB, fat body; SG, salivary glands; MG, midgut. **B.** Immunofluorescence staining to reveal the distribution of LsVg protein in *L*. *striatellus* tissues. LsVg was probed with mouse anti-LsVg monoclonal antibody Ab47Km and stained with Alexa Fluor 568 (shown in red). Nucleoli were stained with TO-PRO-3 (shown in blue). Images were examined using a Leica TCS SP8 confocal microscope. Images are representative of three independent experiments with a total of 15 SBPHs analyzed. The white arrow indicates the immune-reactive signal of the LsVg protein. The scale bar, 50 μm.

Quantitative real-time PCR (qRT-PCR) was performed to test the *LsVg* gene expression levels. Both the fat body and hemocytes produced abundant *LsVg* mRNA. By contrast, only a few of the salivary gland and midgut samples exhibited *LsVg* expression, all at low levels (Fig 1A). An immunofluorescence assay (IFA) was performed to visualize LsVg protein distribution. Using an LsVg-specific monoclonal antibody (designated as Ab47Km in this study) against the polypeptide fragment RNQQKTKSRSRRS [5], the LsVg protein was found to localize in four types of tissue at varying abundances (Fig 1B). Consistent with the observed mRNA levels, LsVg protein was abundantly synthesized in both the fat body and hemocytes (Fig 1B), but was only at the detectable levels in a few of the salivary gland and midgut samples.

Given that multiple *L*. *striatellus* tissues expressed LsVg and that both the fat body and hemocytes produced the protein at high levels, we next investigated whether LsVg from these tissues plays a role in mediating RSV transmission.

### Only hemocyte-produced Vg co-localized with RSV in vivo

**Fig 2:**
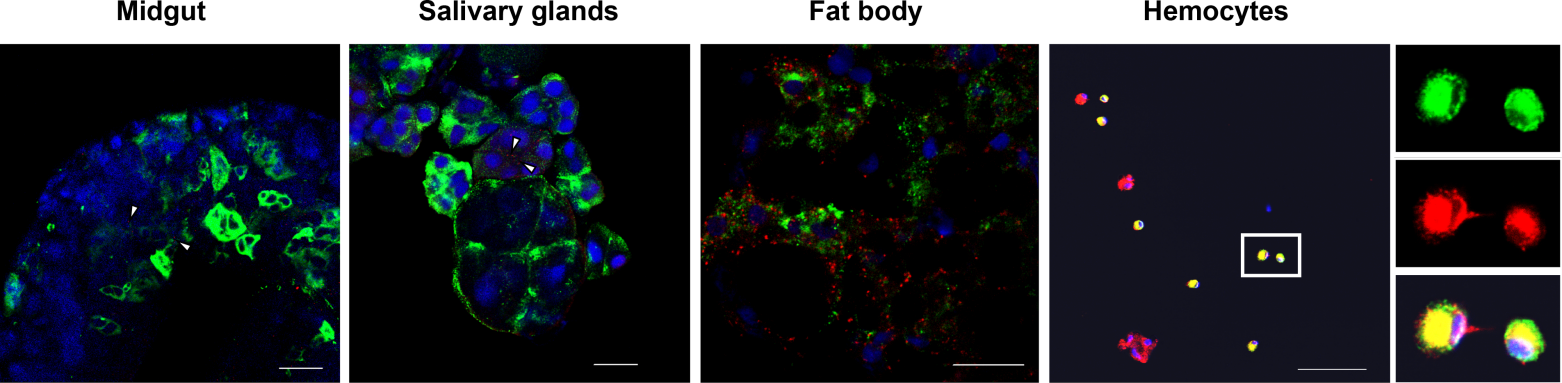
Localization of LsVg and RSV in female *L*. *striatellus* tissues. RSV was probed with Alexa Fluor 488-labeled mouse anti-RSV monoclonal antibody (shown in green). LsVg was probed with mouse anti-LsVg monoclonal antibody Ab47Km and stained with Alexa Fluor 568 (shown in red). Nucleoli were stained with TO-PRO-3 (shown in blue). Images were examined using a Leica TCS SP8 confocal microscope. Images are representative of three independent experiments with a total of 15 SBPHs analyzed. The white arrow indicates the immune-reactive signal of the LsVg protein. The scale bar represents 20 μm.

To determine the tissue or space in which the LsVg–RSV interaction occurred, IFAs were performed to detect the co-localization of LsVg and RSV in the insect tissues. By using antibodies against the RSV RNPs, we found that the virus was distributed in all four tested *L*. *striatellus* tissues (Fig 2). To our surprise, LsVg and RSV co-localized only in hemocytes. Even though LsVg was abundant in the fat body, the protein did not co-localize with RSV in that tissue. No co-localization was observed between RSV and the low levels of LsVg in the midgut or salivary glands either.

Previous studies have demonstrated that the Vg homologous protein is proteolytically cleaved into several subunits before being deposited in the eggs as vitellin (Vn) polypeptides [19]. Our earlier investigation of *L*. *striatellus* Vg also indicated that its N-terminal Vit-N domain does not interact with the RSV CP *in vitro* [5]. We thus hypothesized that LsVg is tissue-specifically processed and that the molecular form existing in the fat body is unable to interact with RSV. To test this hypothesis, we analyzed the LsVg cleavage profile and used subunit-specific anti-LsVg antibodies to investigate the subunit composition of LsVg in different tissues.

### LsVg was proteolytically cleaved to produce four detectable vitellin subunits

Insect Vgs are usually proteolytically cleaved prior to secretion into the hemolymph, whereas the Vg subunits, in some cases, are further processed in the ovaries [17, 19]. To reveal processing patterns of the LsVg protein in different tissues, we first analyzed the subunit composition of the *L*. *striatellus* vitellin (LsVn) and then used subunit-specific antibodies to determine the molecular form of LsVg in particular tissues.

**Fig 3:**
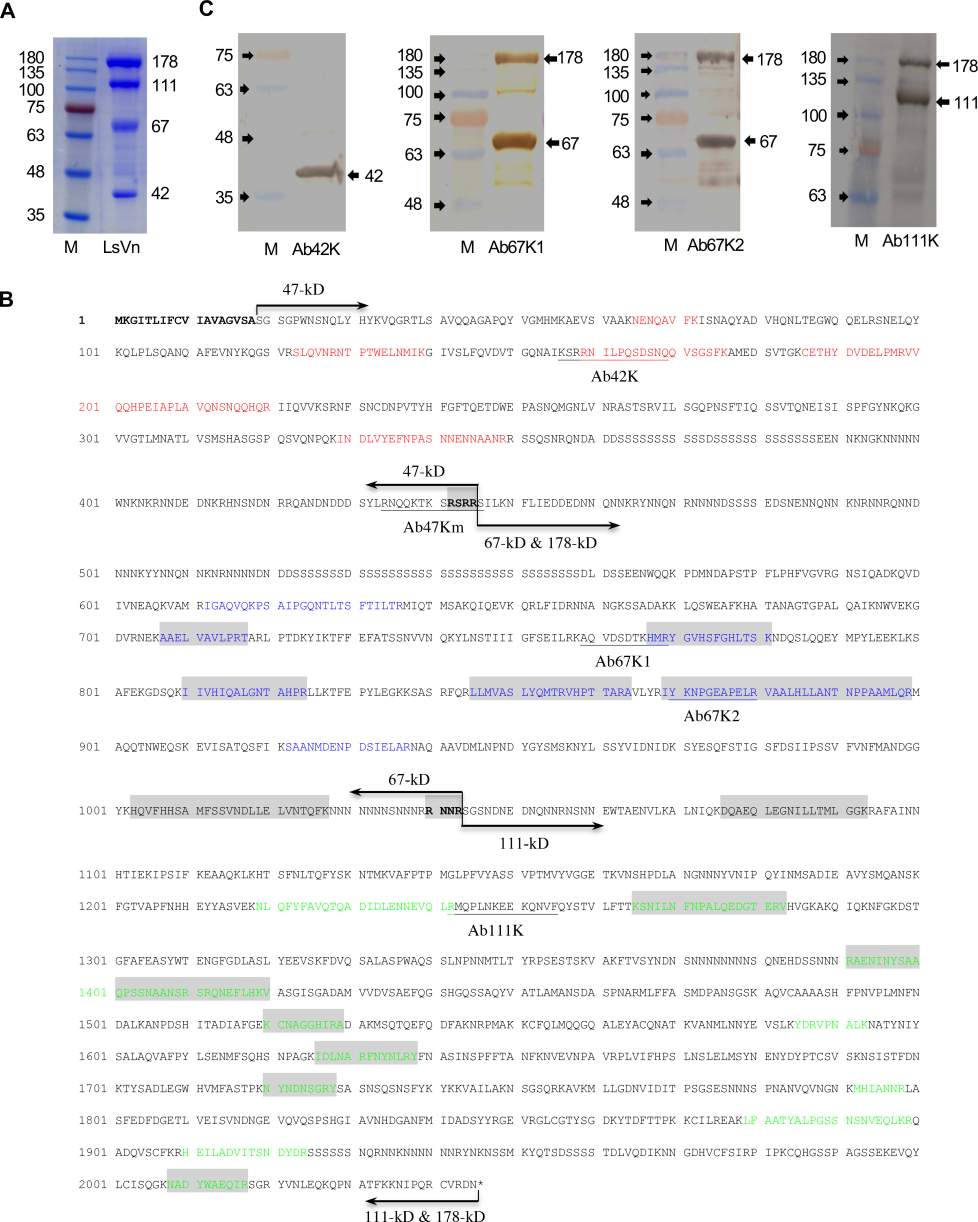
Subunit composition of LsVn. **A.** SDS-PAGE (10%) of purified LsVn. M is the molecular weight marker (kDa). The identified LsVn subunits are indicated by the arrows on the right. **B.** Mapping of vitellogenin-derived peptides identified by mass spectrometry onto the LsVg primary sequence. Peptides identified from SDS-PAGE bands are indicated by color: 178 kDa (shaded), 111 kDa (green), 67 kDa (blue) and 42 kDa (red). Pairs of arrows mark the span of LsVg or LsVn subunits. Shaded tetra-residues in bold font are the cleavage sites. Underlined sequences indicate synthetic peptides used for the production of subunit-specific antibodies. The predicted signal peptide sequence at the N-terminus is shown in bold. **C.** Verification of the composition of the LsVn subunit by western blot analysis. Purified LsVn was fractionated by SDS-PAGE (10%) and probed with the subunit-specific antibodies. Identified LsVn subunits are indicated by the arrows on the right. M, the molecular weight marker (kDa).

LsVn was purified from female insects 3 days after molting, and the LsVn subunits were fractionated by SDS-PAGE. The purified LsVn resolved into four well-separated major bands with molecular sizes of approximately 178, 111, 67 and 42 kDa (Fig 3A). Mass spectrometry was performed to identify the LsVg-derived peptides of the four SDS-PAGE bands. Localization of the identified peptide hits revealed that the 42- and 178-kDa bands corresponded to the N- and C-parts of the LsVg protein, respectively. Peptide hits from the 67- and 111-kDa bands were included in the 178-kDa large subunit, where they clustered on its N- and C-parts, respectively. These results revealed the cleavage profile of LsVg. In particular, the precursor protein is first cleaved into small (42 kDa) and large (178 kDa) subunits, with the latter further cleaved into two medium-sized subunits (67 and 111 kDa). As insect Vgs are usually cleaved at the consensus tetra-residue motif RXXR by subtilisin-like endoproteases [15, 16], the LsVn subunit composition suggested the existence of two potential RXXR cleavage sites.

Inspection of the 2,045 amino acids of the LsVg protein sequence allowed us to identify seven RXXR motifs: RSRR ending at amino acid 445, RNNR at 496, RTAR at 719, RFQR at 844, RNNR at 1,043, RSGR at 2,020 and RCVR at 2,043. Based on ProPeptide cleavage site prediction (ProP 1.0 Server) [36], an above-threshold cleavage score, 0.873, was obtained for the RSRR motif flanked by conserved polyserine domains (Fig 3B). Cleavage after this motif resulted in two subunits with calculated molecular weights of 47 and 178 kDa. Because all the other RXXR motifs had cleavage scores below the prediction threshold, the second cleavage site was determined to be RNNR ending at amino acid 1,043; this inference, which was based on the molecular weight of the LsVn subunits (67 and 111 kDa) as well as on the mass-spectrometry results, was further verified experimentally (see below and Fig 3C). Cleavage at this motif divided the 178-kDa large subunit into two medium-sized subunits with molecular weights of 67 and 111 kDa (Fig 3B).

Peptide-based antibodies were produced for specific recognition of the corresponding LsVn subunits. Antibodies Ab42K and Ab111K were produced based on peptides within the mass-spectrometry-confirmed regions of the 42- and 111-kDa LsVn subunits (Fig 3B). Because multiple RXXR motifs were distributed within the calculated 67-kDa LsVn subunit, two antibodies, Ab67K1 and Ab67K2, were produced to recognize different regions of this fragment (Fig 3B). Western blotting confirmed the predicted RXXR cleavage motifs. As expected, antibody Ab42K recognized the 42-kDa band, both Ab67K1 and Ab67K2 recognized the 67- as well as the 178-kDa bands, and Ab111K exhibited immunoreactive signals to both 111- and 178-kDa bands (Fig 3C). The small LsVn subunit had a molecular weight (42 kDa) lower than the calculated value of 47 kDa, which suggests that further processing might occur in the ovaries. Given that no additional RXXR motif was present within the 47-kDa polypeptide, how this protein processing occurs is unclear.

According to the observed LsVg cleavage pattern, three subunit-specific antibodies (Ab42K, Ab67K2 and Ab111K) were used in the subsequent experiments to analyze the molecular forms of LsVg present in different tissues.

### The fat body mainly harbored the small LsVg subunit

**Fig 4:**
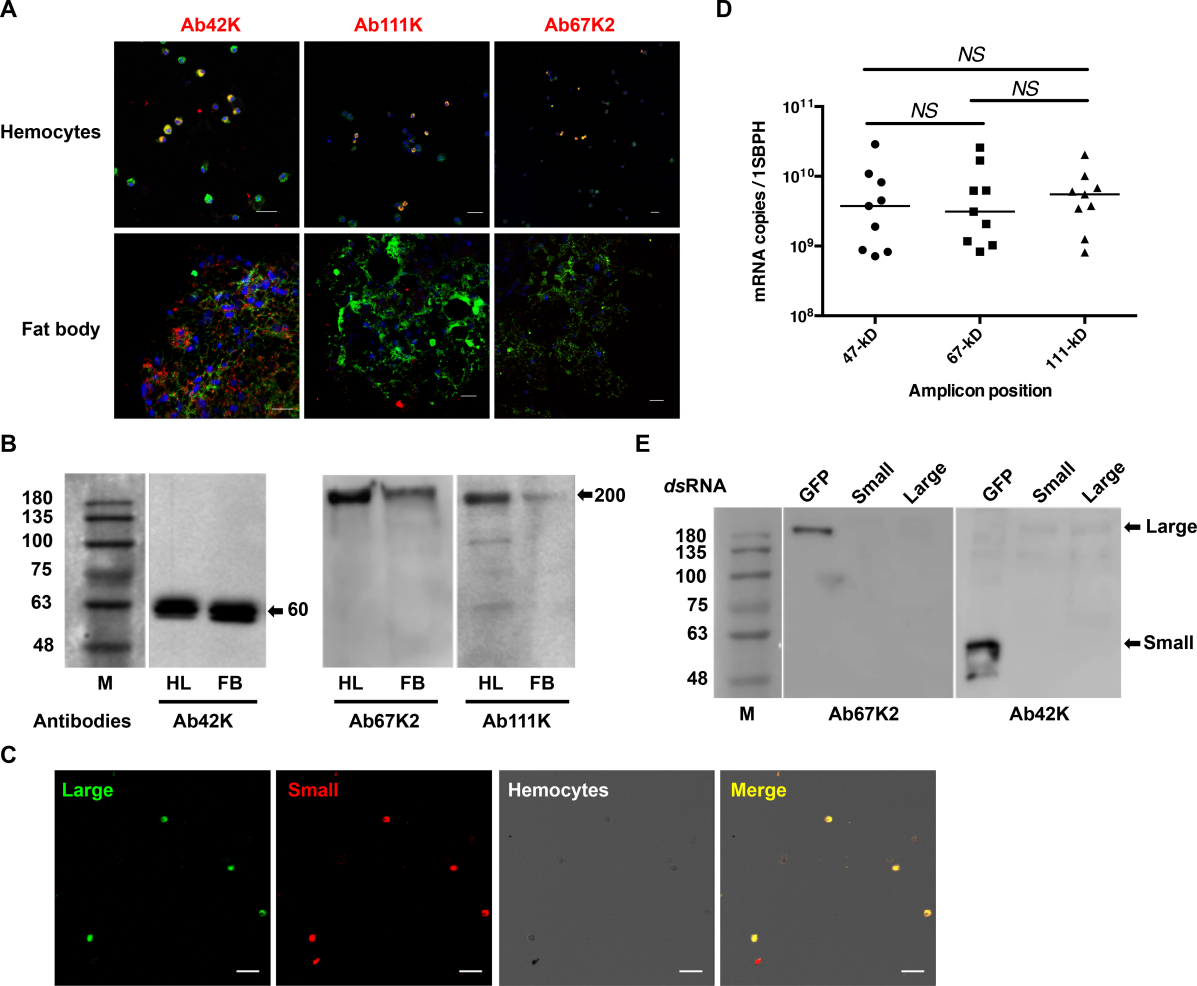
Tissue-specific processing of LsVg in female *L*. *striatellus*. **A.** Confocal microscopy to reveal the distribution of different LsVg protein regions in the fat body or hemocytes. The LsVg N-terminus (recognized by antibody Ab42K) was present in both tissues, whereas the middle region (recognized by Ab67K2) and the C-terminus (recognized by Ab111K) existed only in hemocytes. LsVg probed with the LsVn subunit-specific antibody was stained with Alexa Fluor 568 (shown in red). RSV was stained with Alexa Fluor 488 (shown in green). Nucleoli were stained with TO-PRO-3 (shown in blue). Images were examined using a Leica TCS SP8 confocal microscope. The scale bar represents 20 μm. **B.** Western blots to determine the molecular weights and subunit distribution of proteins in the fat body (FB) or hemolymph (HL). Extracted hemolymph or fat-body proteins were fractionated by SDS-PAGE (10%) and probed with the subunit-specific antibodies Ab42K, Ab67K2 and Ab111K. M is the molecular weight marker (kDa). Identified LsVg subunits are indicated by the arrows on the right. **C.** Confocal microscopic images showing co-localization of the N-terminal small (Small) and C-terminal large (Large) subunits of LsVg. The large subunit was probed with antibody Ab111Km and stained with Alexa Fluor 488 (shown in green). The small subunit was probed with antibody Ab42K and stained with Alexa Fluor 568 (shown in red). Images were examined using a Leica TCS SP8 confocal microscope. The scale bar represents 20 μm. **D.** The mRNA abundance of LsVn subunits. The mRNA copy numbers were determined by SYBR Green-based *q*PCR. Each dot, square or triangle represents one fat-body sample collected from one female SBPH. *NS*, not significant. **E.** Western blots showing the influence of subunit-specific gene silencing on expression levels of multiple subunits. RNAi with *ds*RNA specific to either the N-terminal small (Small) or C-terminal large (Large) subunit dramatically decreased expression levels of both subunits. RNAi with *dsGFP* was used as a negative control and did not influence the expression of LsVg. Protein levels were detected with antibodies Ab67K2 or Ab42K. M is the molecular weight marker (kDa). Positions of the LsVg subunits (Small and Large) are indicated by the arrows on the right.

IFA was performed using subunit-specific antibodies to detect subunit distribution. Similar to the results obtained with antibody Ab47Km (Fig 1B), the use of the Ab42K antibody resulted in immunoreactive signals in both the fat body and hemocytes (Fig 4A). By contrast, Ab67K2 and Ab111K produced immunoreactive signals only in hemocytes (Fig 4A). These results indicated that only the N-terminus of LsVg exists in the fat body. Western blotting was performed to determine the molecular weights and the subunit composition of proteins in the fat body extract and hemolymph. Using the Ab42K antibody, a 60-kDa band was detected in both the fat body extract and the hemolymph (Fig 4B). Antibodies Ab67K2 and Ab111K both recognized a strong band of approximately 200 kDa in the hemolymph (Fig 4B) as well as a similar but much weaker band in the fat-body extract (Fig 4B). As no full-length LsVg protein was recognized by any of the three antibodies, we hypothesized that the LsVg protein, when synthesized, undergoes rapid cleavage to yield two subunits with molecular sizes of 60 and 200 kDa. While both subunits exist inside the hemocytes, only the small 60-kDa subunit exists in the fat body. The weak 200-kDa band detected by western blotting with LsVg C-terminal antibodies (Ab67K2 and Ab111K) in fat-body extracts may represent unconsumed proteins, or contamination from the hemolymph attached to fat-body tissues. The detected 60 and 200 kDa bands were larger than their calculated sizes (47 and 178 kDa); this increased molecular size may be due to post-translational modifications such as glycosylation, lipidation, phosphorylation or sulfation [12, 13]. These results also suggested that LsVg was cleaved at a single site (RSRR) prior to protein invasion of the ovaries. We then performed coimmunoprecipitation assay to confirm the *in-vivo* physical interaction between RSV and the LsVg subunits. The anti-RSV antibodies coimmunoprecipitated the LsVg large subunit, but not the small one, from the female crude extracts, confirmed the physical interaction occurred between RSV and the large LsVg subunit (S1 Fig).

We then performed dual immunofluorescence assay to analyze the distributional pattern of the two LsVg subunits within hemocytes. Hemocytes were probed with two different antibodies: Ab111Km antibody followed by staining with the fluorescence dye Alexa 488, or with Ab42K antibody stained with Alexa 568. The two types of fluorescence signals were merged in all LsVg-expressing hemocytes (Fig 4C), indicating the co-existence of N- and C-terminal LsVg subunits within individual hemocytes. These results, combined with the protein sizes revealed by western blotting, indicated that LsVg exists in hemocytes as a complex of large and small subunits.

To determine whether the full-length mRNA transcript or a truncated version is expressed in the fat body, we designed three pairs of PCR primers to amplify the different regions of *LsVg*. qRT-PCR analysis indicated that the three mRNA fragments were expressed at similar abundances (Fig 4D), suggesting the production of the full-length transcript. We also used RNA interference (RNAi) with double-stranded RNA of the large LsVg subunit to knockdown gene expression, and then measured the expression level of the small subunit. Western blotting with antibodies Ab42K and Ab67K2 revealed dramatically decreased LsVg protein levels following gene knockdown (Fig 4E), indicating a single transcript containing both the small and large subunits. We also cloned the full-length *LsVg* mRNA from both the hemocytes and fat body of female SBPHs. Comparison of the LsVg cDNA sequences from these tissues did not detect any variable splicing.

Taken together, our results indicated that the LsVg protein is expressed in both hemocytes and the fat body of *L*. *striatellus*. After synthesis, the protein is cleaved at the RSRR motif, resulting in large and small subunits. The two-subunit complex exists in hemocytes, whereas only the small subunit remains in the fat body. How and why the large LsVg subunit is depleted in the fat body remains unclear.

### LsVg was expressed by nymphs and males, in hemocytes only, and co-localized with RSV

**Fig 5:**
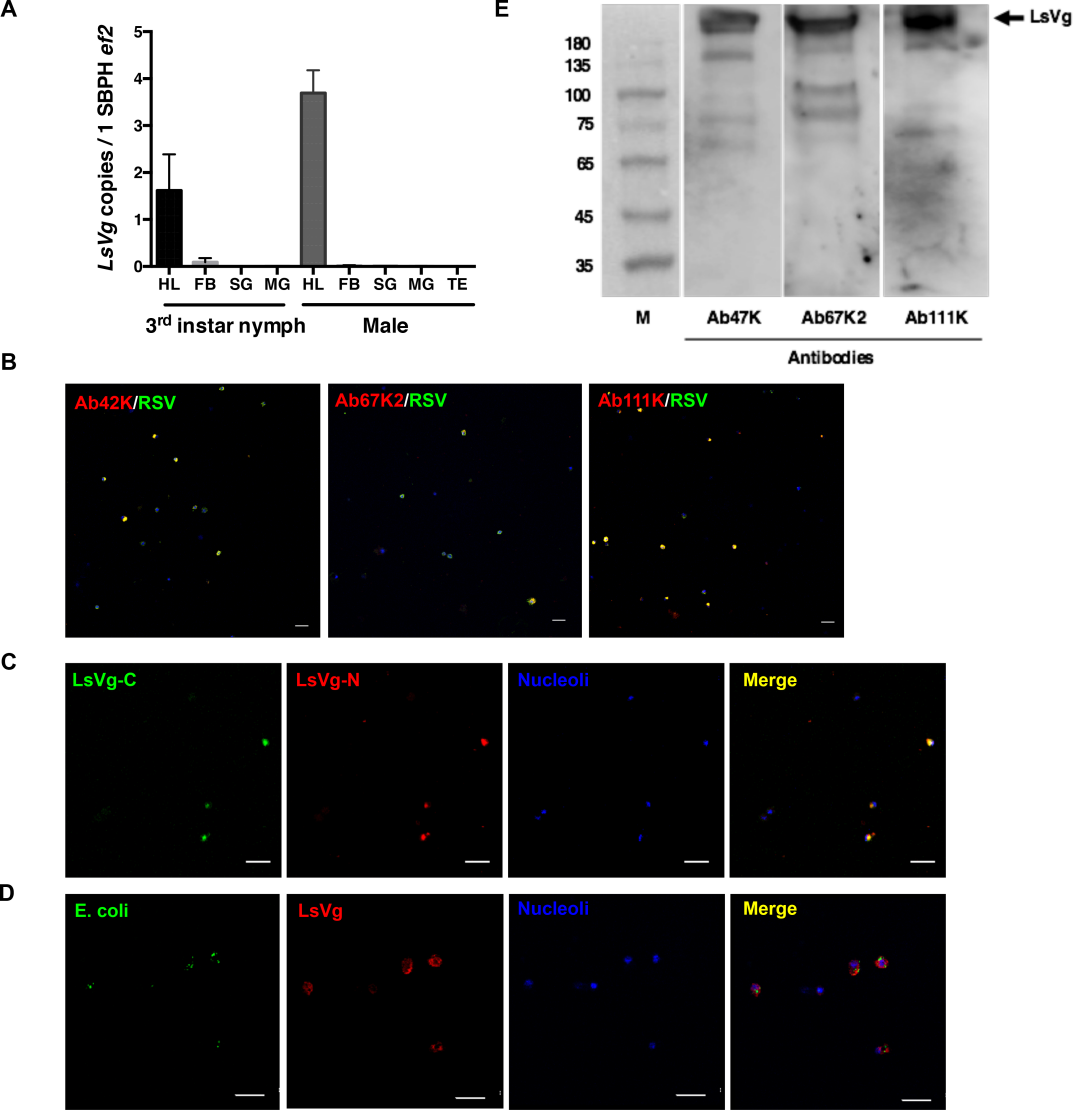
LsVg expression in SBPH nymphs and males. **A.** *LsVg* mRNA distribution in different tissues of SBPH nymphs and males was revealed by qPCR. The mean and SD were calculated from three independent experiments, with four mRNA samples per experiment. Ef2, *L*. *striatellus* elongation factor 2 gene; HL, hemolymph; FB, fat body; SG, salivary glands; MG, midgut. TE, testis. **B.** Confocal microscopic image showing the existence of LsVg and its co-localization with RSV in hemocytes. LsVg was probed with LsVn-subunit specific antibodies Ab42K, Ab67K2 and Ab111K and stained with Alexa Fluor 568 (shown in red). RSV was stained with Alexa Fluor 488 (shown in green). Nucleoli were stained with TO-PRO-3 (shown in blue). **C.** Confocal microscopic image showing co-localization of the N-terminal small (Small) and C-terminal large (Large) subunits of LsVg. The large subunit was probed with antibody Ab111Km and stained with Alexa Fluor 488 (shown in green). The small subunit was probed with antibody Ab42K and stained with Alexa Fluor 568 (shown in red). **D.** Confocal microscopic image showing localization of LsVg and phagocytosed *E*. *coli* containing the *gfp* gene conferring green fluorescence. LsVg was probed with Ab42K and stained with Alexa Fluor 568 (shown in red). Images were examined using a Leica TCS SP8 confocal microscope. The scale bar represents 20 μm. **E.** LsVg is not cleaved in male *L*. *striatellus*. Extracted hemolymph proteins were fractionated by SDS-PAGE (10%) and probed with the subunit-specific antibodies Ab47Km, Ab67K2 and Ab111K. M, the molecular weight marker (kDa). Arrows on the right, identified LsVg proteins.

In addition to nourishing developing embryos, insect hemocytes and hemolymph play key roles in innate immunity. We thus investigated whether LsVg is also expressed by hemocytes of nymphal or male *L*. *striatellus*. According to a qRT-PCR analysis, both the nymphs and males expressed *LsVg*, and hemocytes were the only *LsVg*-expressing tissue (Fig 5A). An IFA using the three subunit-specific antibodies (Ab42K, Ab67K2 and Ab111K) further confirmed the qRT-PCR results. All antibodies reacted to the corresponding subunits in hemocytes (Fig 5B). A dual immunofluorescence experiment with Ab42K and Ab111Km was used to reveal co-localization of the large and small subunits (Fig 5C). When expressed in hemocytes, the LsVg protein co-localized with RSV (Fig 5B). To exclude the possibility that LsVg bound to various pathogens in a non-specific manner, we microinjected *Escherichia coli* into the *L*. *striatellus* hemolymph and looked for the localization of the LsVg protein with the phagocytosed bacteria. The bacteria did not co-localize with LsVg inside hemocytes (Fig 5D), indicating that the interaction between RSV and LsVg was a specific event.

Western blotting was performed to determine the subunit composition of LsVg in the male hemocytes. All the three antibodies Ab47K, Ab67K2 and Ab111K recognized a strong band of more than 200 kDa in the hemolymph (Fig 5E), indicating that the LsVg in male remained in its full-length molecular form.

### LsVg deficiency decreased the RSV titers in the hemolymph

**Fig 6:**
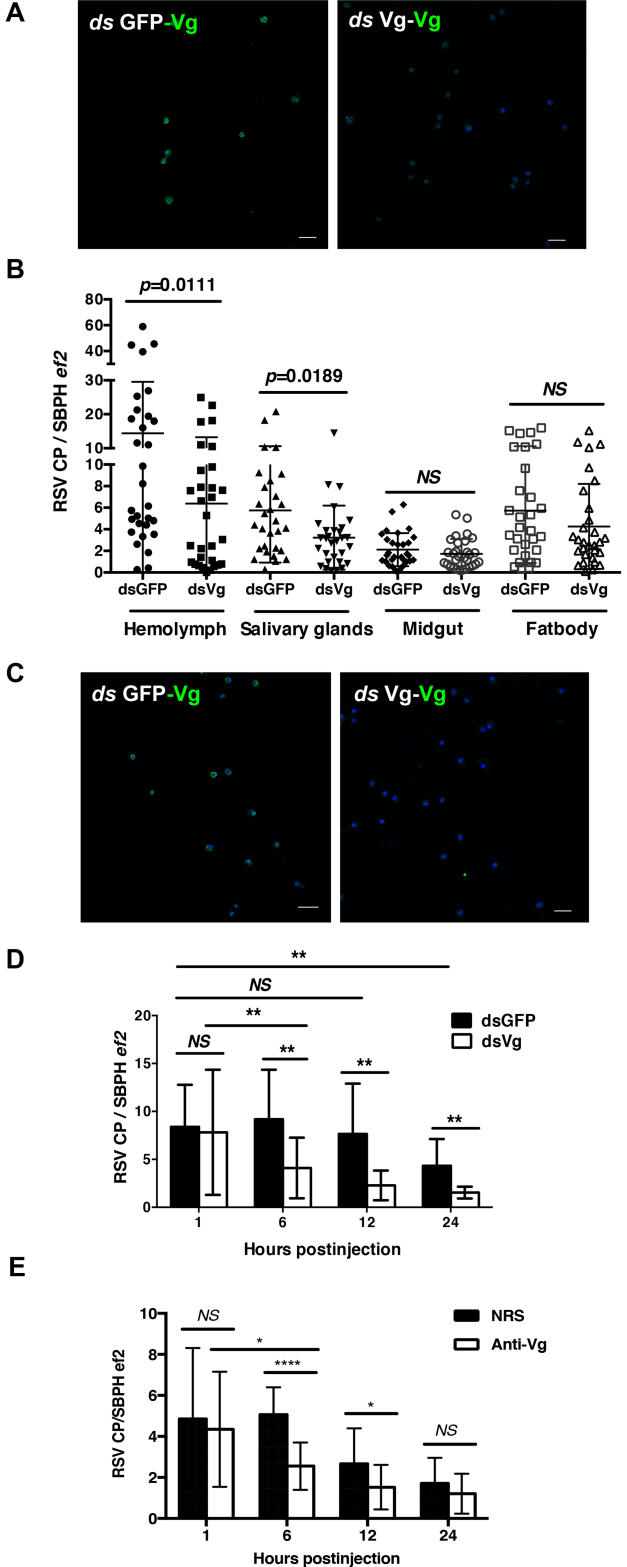
Influence of LsVg deficiency on RSV survival and transmission. **A and C.** Treatment of RSV-infected (A) and RSV-free (C) three-instar nymphs with the *ds*RNA of LsVg (*ds*Vg), which resulted in significantly lower LsVg expression levels compared with those after treatment with the *ds*RNA of GFP (*ds*GFP). LsVg was probed with antibody Ab111Km and stained with Alexa Fluor 488 (shown in green). Nucleoli were stained with TO-PRO-3 (shown in blue). Images were examined using a Leica TCS SP8 confocal microscope. The scale bar represents 20 μm. **B.** In RSV-infected SBPHs, LsVg-deficiency decreased the RSV titer in both the hemolymph and salivary glands but had no effect in the midgut or fat body. **D.** In RSV-free SBPHs, RSV delivered into *ds*Vg-treated insects exhibited significantly decreased survival rates compared with those of dsGFP-treated insects following virus delivery. **E.** Influence of anti-Vg antibodies on RSV survival in *L*. *striatellus* hemolymph. NRS, normal rabbit serum. *NS*, not significant. **, *p*<0.01, *, *p*<0.05, ****, *p*<0.0001. The mean and SD were calculated from three independent experiments. CP, the RSV capsid protein; *ef2*, the *L*. *striatellus* elongation factor 2 gene.

To determine the role of LsVg in mediating RSV survival and transmission within the insects, we generated *LsVg*-deficient *L*. *striatellus* nymphs using RNAi with *ds*LsVg via microinjection. At day 6 following the onset of feeding, LsVg expression levels within hemocytes analyzed by IFA using antibody Ab111Km exhibited a dramatic reduction (Fig 6A). We then assessed whether RSV transmission was affected by RNAi-mediated *LsVg* deficiency. The RSV titers in various *L*. *striatellus* tissues were assessed by qRT-PCR. Compared with the control group injected with *ds*GFP, *LsVg ds*RNA-treated *L*. *striatellus* showed similar RSV titers in the midgut and fat body, whereas significantly lower virus titers were observed in the hemolymph and salivary glands (Fig 6B). These results indicate that LsVg functions in facilitating RSV survival in, and transmission through the hostile hemolymph environment.

To confirm the function of LsVg in facilitating RSV survival in the hemolymph environment, equal volumes of *LsVg* or *GFP ds*RNA were delivered into the hemocoel of RSV-free *L*. *striatellus* nymphs via microinjection. Insects were allowed to feed on RSV-free rice seedlings. At 72 h following the microinjection, when the *LsVg* expression levels had been dramatically reduced by *dsLsVg* treatment (Fig 6C), purified RSV RNPs were directly delivered into the insect hemocoel. At 1, 6, 12 and 24 h following virus delivery, RSV titers in the *L*. *striatellus* body were assessed by qRT-PCR. Compared with the control group treated with *GFP ds*RNA, *LsVg ds*RNA-treated *L*. *striatellus* showed significantly lower virus titers beginning 6 h post-injection (Fig 6D). These results further confirmed that the hemocyte-produced LsVg plays a role in facilitating RSV survival in the hostile hemolymph environment.

Immuno-blocking experiment with anti-Vg antibodies revealed similar results. Inoculation of anti-Vg antibody into hemolymoh before RSV delivery decreased RSV survival in the hemolymph, especially at the early stages (Fig 6E, 6h and 12 h); however, the difference was not significant at the later stage (Fig 6E, 24 h). It might be that most of the RSVs were inside the hemocytes at the late stage, so immuno-blocking did not affect the function of the intracellular Vg.

## Discussion

Plant viruses have been reported to achieve vertical transmission within insect vectors via the transovarial transportation system of the insect Vg protein [5, 37]. Traditionally, insect Vg transported into the ovaries has been thought to be synthesized in the fat body. In the current study, we demonstrated that *L*. *striatellus* hemocytes also synthesize abundant Vg protein and that only hemocyte-produced Vg interacts with RSV *in vivo*. By clarifying the subunit composition of LsVn and by using LsVn subunit-specific antibodies, we revealed that LsVg is synthesized and proteolytically cleaved into the N-terminal small subunit and the C-terminal large subunit in both the fat body and hemocytes. The large subunit produced in the fat body is further consumed, with the large subunit that remains in hemocytes capable of interacting with the RSV CP. Moreover, we showed that LsVg is also expressed in male and nymphal SBPHs and revealed the gender-independent function of LsVg. LsVg expressed in the hemocytes of non-female SBPHs can also interact with RSV *in vivo*, thus protecting the virus from the hostile hemolymph environment and facilitating its systemic infection.

This study identified hemocytes, a major component of the insect immune system, as a new Vg-producing tissue. To our knowledge, this is the first study to demonstrate that an insect *Vg* gene is expressed by hemocytes. We also addressed the function of the hemocytes-produced Vg protein in virus recognition and transmission. The insect *Vg* gene has been previously reported to be expressed in tissues other than the female fat body. Although the Vg protein was not functionally investigated in most of these studies, its action has been physiologically elucidated in some cases. For example, *Apis mellifera* Vg is synthesized in the hypopharyngeal glands and the adjacent head fat-body cells of functionally sterile honeybee workers, implying that Vg is used for brood food production [38–40]. In *Bombus hypocrita*, the *Vg* gene is expressed at various levels in different castes, including the queen, workers and drones, from pupal to adult stages [41]. *Camponotus festinatus* Vg has been identified in both the queen and workers. The concentration of Vg is higher in queenless workers than in queenright ones, and Vg is synthesized at low concentrations before adult eclosion [42]. In addition to tissue-specific expression, tissue-specific processing of the Vg protein has been reported. For example, *Leucophaea maderae* Vg is expressed in both the male and female fat bodies. In this species, Vgs produced in the two sexes are similar in terms of their native molecular weights, but differ in their cleavage profiles and polypeptide compositions [14, 43]. At present, little is known about the relationship between the function of Vg and its biochemical and structural properties. By addressing the functional relationship determined by the LsVg subunit/domain composition, we have confirmed the possibility that proteolytically processed Vg with different domains can mediate specific functions. To our knowledge, our investigation is one of the few studies to have classified the function of Vg according to its subunit or domain composition.

The mature Vg contains an N-terminal domain (Vitellogenin_N [Vit_N)], a middle-region domain of unknown function (DUF1943), and a von Willebrand factor type D (vWD) C-terminal domain [5, 33, 44–46]. Domain Vit_N is required for interaction with the Vg receptor (VgR) [9, 47], while domains DUF1943 and vWD play roles in pathogen recognition [48]. An example in vertebrate animals is that of *Oreochromis aureus* Vg, which interacts with VgR via a polypeptide fragment located in the Vit_N domain [9]. Arthropoda, *Macrobrachium rosenbergii* Vg also interacts with its receptor via a specific *β*-sheet region in the Vit_N lipoprotein domain [47]. In scallops, both the recombinant DUF1943 and vWD domains of the *Patinopecten yessoensis* Vg protein can interact with the lipopolysaccharides and lipoteichoic acid expressed on the bacterial cell wall [45]. In insects, the *L*. *striatellus* Vg protein has been well studied by *in vitro* experiments that have revealed strong, weak and non-existent interactions between the RSV CP and vWD, DUF1943 and Vit_N domains, respectively [5]. In this study, we verified that the fat body-produced N-terminal Vg subunit is included in the Vit_N domain sequence. Because the *L*. *striatellus* fat body-Vg lacks any microbe-binding domains, it plays no role in mediating RSV transmission. This subunit contains both a signal peptide for secretion and a recognition site for receptor binding, however, and is thus expected to be secreted and then taken up by oocytes.

Our analysis revealed the existence of an N-terminal LsVg fragment—in contrast to the complete protein—in abdominal fat body tissue. Three lines of evidence demonstrate that the N-terminal LsVg fragment is processed from the full-length LsVg protein in the fat body rather than being synthesized from a different gene. Firstly, the polyserine tracts flanking the R-X-R/K-R motif have been demonstrated to be cleaved in almost all Vg homologs (see review [7]). Second, qRT-PCR analysis detected similar mRNA abundance levels between N- and C-termini of the *Vg* gene (Fig 4D). Third, and most importantly, *ds*RNA of the C-terminal large subunit dramatically interfered with mRNA levels of the N-terminal small subunit (Fig 4E). The significance of the existence of this Vg fragment in the fat body remains unclear; however, this type of Vg processing has also been reported in honeybees [23]. Havukainen *et al*. have presented a structural model for the N-terminal Vg fragment that includs a conserved *β*-barrel-like shape, with a lipophilic cavity and two insect-specific loops, thus indicating a capacity for lipid transport.

In general, arthropod hemolymph is hostile to pathogens because it contains both circulating hemocytes and antimicrobial proteins [49–51]. Viruses acquired via the insect midgut can accordingly be transported to other tissues only after successful escape from the hostile hemolymph environment (e.g., through survival in plasma and evasion of hemocyte phagocytosis). Studies have revealed, however, that a successfully transmitted microbe can protect itself against the host immune system by exploiting host hemolymph proteins, thereby transforming the host hemolymph into a relative benign environment. Examples include Potato leafroll virus transmission by the green peach aphid *Myzus persicae* and Tomato yellow leaf curl virus transmission by the sweetpotato whitefly *Bemisia tabaci*. In these cases, the viruses gain protection and achieve successful transmission by binding to GroEL, a hemolymph protein produced by endosymbiotic bacteria of the insect [52–54]. During the transmission of West Nile Virus by *Aedes aegypti*, viruses can even establish infection in hemocytes by binding to the mosquito hemolymph protein that leads to the phagocytosis pathway [55]. In previous studies, Vg from fish has been revealed to have immunologic functions. By binding to the pathogen, the Vg protein can neutralize/kill the pathogen, either directly or in an indirectly fasion as opsonins mediate macrophage phagocytosis [26, 27, 33]. However, whether recognition by Vg of an arthropod vector would result in virus tolerance is not clear. In this study, the *L*. *striatellus* hemolymph was revealed to be a relatively benign environment for RSV. RSV tends to be cleared from the SBPH hemolymph slowly. When *E*. *coli* was delivered into the *L*. *striatellus* hemolymph through microinjection, the maximum level of phagocytosis was reached at 1.5–2 h post injection, and the bacterial numbers in the hemolymph (including in hemocytes) were dramatically decreased to <20% within 24 h (S2 Fig). When RSV suspension was delivered into the hemolymph, by contrast, maximum phagocytosis was reached at about 20 h post-injection (S2 Fig), and 50% of the virus was retained in the hemolymph (including the hemocytes) after 24 h (Fig 6D). By uncovering a positive correlation between the Vg presence and the RSV in-hemolymph survival (Fig 6), this study has provided clues regarding the contribution of the RSV-LsVg interaction to the protection of RSV in the hemolymph and the development of a benign *L*. *striatellus* hemolymph environment during RSV transmission. This vector-Vg-virus interaction may be a common molecular mechanism to facilitate the passage of the virus through the vector hemolymph.

In summary, we have revealed that an insect Vg protein undergoes tissue-specific processing, with the molecular form produced specifically by the hemocytes used by the virus to aid its survival in the hemolymph, thus facilitating virus transmission. Detailed analyses of the molecular interactions between the virus and its insect vector are required for the exploitation of novel virus control strategies that target specific points in the virus life cycle and interfere with virus transmission.

## Materials and methods

### Viruses, SBPHs and host plants

RSV-free and RSV-infected *L*. *striatellus* individuals used in this study were originally captured in Jiangsu Province, China, and were maintained in our laboratory. All plants used for *L*. *striatellus* rearing were grown inside a growth incubator at 25°C under a 16-h light/8-h dark photoperiod. To ensure a high offspring infection rate, viruliferous female imagoes were cultured separately, 15% of their corresponding offspring were tested for RSV infection through a dot-enzyme-linked immunosorbent assay using RSV-specific monoclonal antibodies provided by Dr Xueping Zhou (Institute of Biotechnology, Zhejiang University [56]). The RSV antibody was produced using the RSV RNPs as antigen. This virion-specific monoclonal antibody was used in all the *in vivo* experiments that were performed to determine RSV localization or co-localization with LsVg. An insect population with a predicted infection rate of 100% was used in the experiments.

### Tissue collection

For hemolymph isolation, the SBPHs were anesthetized at -20 centigrade for 3 min, and then the forelegs were severed at the coxa-trochanter joint by forceps. The hemolymph was expelled and drawn to the tip of clean forceps. Only clear droplets were collected to avoid contamination by fat body [55]. In droplets contaminated by fat body, transparent oil drops can be seen. The SBPHs were then dissected in pre-chilled PBS buffer. Insects were dissected from the abdomen, and the wound was rinsed gently two times with PBS buffer. Because the insect fat body compose of a meshwork of loose lobes suspended in the hemocoel and bathed in the insect hemolymph, it is difficult to dissect it out in its entirety and without contamination of hemolymph or other tissues. We collect most fat body without contamination from other tissues, and placed it in PBS buffer. Tissues including the midgut, salivary glands, ovaries of the female and testes of the male were washed twice in PBS to remove contaminating viruses or proteins from the hemolymph.

### qRT-PCR

For quantitative analysis of LsVg expression in SBPH tissues, we dissected female, male or third-instar nymphal *L*. *striatellus* and collected the tissues according to the protocol described above. RNA was extracted from the tissues of individual insects. Reverse transcriptional PCR and SYBR-Green-based *q*PCR were performed according to the protocols provided by the manufacturer. Primer pairs used to amplify LsVg and LsVn / LsVg subunits were LsVg-QF / LsVg-QR, 47K-QF / 47K-QR, 67K-QF / 67K-QR and 111K-QF / 111K-QR (S1 Table). Viral RNA copies were measured by qRT-PCR using primer sequences pc3-F and pc3-R (S1 Table), which were designed and synthesized according to the nucleocapsid protein (Pc3 or CP) gene sequence (DQ333944). *L*. *striatellus elongation factor 2 (ef2)* was amplified as an internal control for the loading of cDNA isolated from different samples. Primers used for *ef2* amplification were ef2-QF / ef2-QR (S1 Table). Water was used as a negative control.

### Antibody preparation

Mouse anti-Vg monoclonal antibodies against Vg peptides RNQQKTKSRSRRS and RMQPLNKEEKQNVF were prepared by Abmart (Shanghai, China) as previously described [5], and were designated as Ab47Km and Ab111Km in this study. To prepare LsVn subunit-specific antibodies, the LsVn subunit-specific peptides KSRRNILPQSDSNQ, AQVDSDTKHMR, YKNPGEAPELR and RMQPLNKEEKQNVF were conjugated to mcKLH and injected into rabbits, and the corresponding antiserums were prepared by GenScript (Nanjing, China). The antibodies produced were designated as Ab42K, Ab67K1, Ab67K2 and Ab111K respectively.

### Confocal microscopy

Insects tissues were placed in PBS on silylated glass slides (Sigma cat. no. S4651; St. Louis, MO, USA) and allowed to dry. Tissues were then fixed in 4% paraformaldehyde at room temperature for 30 min. The slides were rinsed twice with PBS and then incubated in PBST/FBS (PBS containing 2% Tween 20 and 2% fetal bovine serum) for 30 min. To detect LsVg localization in different tissues, the slides were incubated with mouse anti-Vg monoclonal antibody Ab47Km (1:300 dilution in PBST/FBS) for 1 h and then Alexa 568-labeled goat anti-mouse antibody (1:200 dilution in PBST/FBS) for 1 h. The slides were rinsed three times with PBST, and the nucleoli were stained with TO-PRO-3 iodide (Invitrogen cat. no. T3605; Carlsbad, CA, USA) at room temperature for 3 min. The samples were examined using a Leica TCS SP8 confocal microscope.

To detect co-localization of LsVg with RSV in the SBPH tissues, slides were prepared according to the protocol described above. The anti-RSV monoclonal antibody was labeled with Alexa Fluor 488 according to the Alexa Fluor 488 Monoclonal Antibody Labeling kit (Invitrogen) instructions. The slides were sequentially incubated for 1 h each with antibody Ab47Km (1:300 diluted in PBST/FBS), Alexa 568-labeled goat anti-mouse antibody (1:200 diluted in PBST/FBS) and with Alexa 488-labeled anti-RSV monoclonal antibody and then stained with TO-PRO-3 iodide for 3 min.

To detect co-localization of LsVn subunits with RSV, slides were prepared as described above. The slides were incubated with mouse anti-RSV and rabbit anti-LsVn antibodies (Ab42K, Ab67K2 or Ab111K; 1:1000 dilution in PBST/FBS), followed by Alexa 488-labeled goat anti-mouse and Alexa 568-labeled goat anti-rabbit antibodies (1:200 dilution in PBST/FBS), and finally with TO-PRO-3 iodide for nucleolus staining. To detect co-localization of LsVg subunits in hemocytes, Ab42K and Ab111Km were used as the primary antibodies, and Alexa 568-labeled goat anti-rabbit and Alexa 488-labeled goat anti-mouse antibodies were used as the secondary antibodies. To detect co-localization of LsVg with *E*. *coli* in hemocytes, the GFP-expressing bacteria were suspended in sterile water at an OD_600_ of 1.0. Subsequently, 13.8 nl of the bacterial suspension was delivered into the hemocoel of third-instar nymphs; 1.5 h after microinjection, the insects were dissected and the hemolymph was collected. Slides for confocal microscopy were prepared as described above. The primary antibody for LsVg detection was Ab42K and staining was performed with Alexa 568.

### Vitellin purification and vitellogenin peptide identification by mass spectroscopy

Newly emerged female SBPHs were allowed to grow for 3 days before extraction of Vn. Two grams of the insects were ground in liquid nitrogen into a fine powder and incubated in 1 ml of 0.4 M NaCl solution for 20 min at 4°C. The suspension was centrifuged at 3,300×*g* for 10 min at 4°C to remove insect debris. The supernatant was centrifuged three times at 1,000×*g* for 10 min at 4°C to remove lipid on the surface of the supernatant. The sample was then treated three times as follows: after addition of 8 ml of ddH_2_O, the mixture was incubated overnight at 4°C followed by centrifugation (1,000×*g*, 20 min, 4°C) to precipitate the Vn protein. The precipitated protein was dissolved in 1 ml of 0.4 M NaCl solution and centrifuged again (3,300×*g*, 10 min, 4°C) to remove any undissolved precipitate. The final supernatant was applied to a size-exclusion Superdex 200 10/300 GL column (GE Healthcare, Piscataway, NJ, USA), and the fractions containing Vn of the highest concentration and purity were collected.

For mass spectroscopy analysis, the purified Vn protein was separated on a 10% SDS-PAGE gel (Bio-Rad Laboratories, Hercules, CA, USA) and stained with Coomassie Blue (Bio-Rad). The bands corresponding to the 178-, 111-, 67- and 42-kDa Vn subunits were excised and digested, and the peptides were subjected for liquid chromatography-tandem mass spectrometry analysis. Peptides were identified using an LTQ-Orbitrap XL with Easy nLC-1000 (Thermo Fisher Scientific), and proteomics data were analyzed using Proteome Discoverer 1.4 (Thermo Fisher Scientific).

### Western blot analysis

To confirm the subunit composition of LsVn, the purified Vn protein was fractionated on a 10% SDS-PAGE gel (Bio-Rad) and processed for immunoblotting. The LsVn/LsVg subunit-specific antibodies Ab42K, Ab67K1, Ab67K2 or Ab111K (1:10,000 dilution in PBST/FBS) was used to probe the corresponding LsVn subunits. The bound antibodies were detected by using horseradish peroxidase-conjugated goat anti-rabbit secondary antibodies (Sigma), and the blots were developed using the enhanced chemiluminescence Western Blotting Detection System (GE Healthcare).

Western blotting was performed to measure the subunit composition and molecular sizes of LsVg in the fat body and hemocytes. Both fat body and hemolymph were dissected from the female insects (3 days after molting). Tissues from 50 insects were placed in 100 μl of PBS buffer and boiled in SDS-PAGE loading buffer. When the same amounts of total proteins were loaded to the gel, we found that LsVg was cleaved into two subunits, one small subunit of 60 kDa and one large of 200 kDa; however, the concentration of the two subunits in different tissues were very different (S3 Fig). It was difficult to determine whether the large subunit existed in the fat body or whether the small subunit was secreted from the fat body. We then adjusted the western blotting loading with same amount of the LsVg small subunit as a control, and compared the amounts of the large subunit to determine subunit distribution. We performed western blotting with Ab47Km to determine the amounts of the small subunit in fat body and hemolymoh. Then fat body and hemolymph protein samples with the same amount of the small subunit were applied to the SDS-PAGE, and probed with antibodies Ab42K, Ab67K2 or Ab111K.

### RNA interference

Two DNA fragments, one specific to the coding sequences of the LsVg N-terminal small subunit and the other to the C-terminal large subunit, were PCR amplified and designated as VgN and VgC, respectively. Primer pairs used for the amplification of the VgN and VgC fragments were VgN-si-F/VgN-si-R and VgC-si-F/VgC-si-R, respectively (S1 Table). *Ds*RNA was synthesized using a commercial kit (Ambion) and purified by phenol:chloroform extraction and isopropanol precipitation. Finally, 36.8 nl of *ds*RNA at 1 ng/nl was delivered into the insect hemocoel for gene silencing. *GFP ds*RNA, which was used as a negative control, was synthesized and microinjected following the same protocol.

To confirm that both LsVg subunits were expressed from the same transcript, *ds*RNA of VgC was delivered into the hemocoel of the fifth-instar nymphal *L*. *striatellus* individuals. The insects were cultured in new chambers with healthy rice seedlings until emergence of adults. Females were transferred to new chambers for an additional 48 h of culture. Then the insects were dissected, and the fat body protein extracts were prepared for Vg expression analysis. Western blotting was performed according to the procedure described above. The antibodies used for the detection of the fat body-expressing LsVg subunit were Ab42K and Ab67K2.

To determine the influence of LsVg on RSV horizontal transmission, *ds*RNA of VgC was delivered into the hemocoel of RSV-infected third-instar nymphs of *L*. *striatellus*. On day 6 of culture in new chambers, a subset of the insects was collected and the expression of Vg in their hemocytes was measured by confocal microscopy. The remaining insects were collected, and the RSV titers in various tissues were measured. Tissues, including the hemocytes, midgut, salivary glands and fat body, were collected as described above. Virus titers were determined by qRT-PCR using primer pair pc3-F / pc3-R (S1 Table).

To determine the effect of LsVg on RSV survival inside the hemolymph, *ds*RNA of VgC was delivered into the hemocoel of RSV-free third-instar nymphal *L*. *striatellus* individuals. After 72 h of culture in new chambers, some of the insects were collected, and the expression of Vg in their hemocytes was measured by confocal microscopy. Following successful knockdown of Vg expression, purified virus RNPs in PBS buffer were microinjected into the insect hemocoel. At 1, 6, 12 or 24 h after RSV microinjection, RNA was extracted from the whole insect body and virus titers were measured according to the protocol described above.

### Statistical analyses

All graphing and statistical analyses were performed using Prism 6.0 software (GraphPad Software, CA, USA). Data were expressed as means ± standard deviation (SD). The significance of differences between groups was evaluated using Student’s t-test.

## Supporting information

S1 Table Primers used in this study. (PDF)

S1 Fig Coimmunoprecipitation (Co-IP) of vitellogenin (Vg) with anti-RSV antibody in the crude extracts of female SBPHs. H, RSV-free SBPHs; I, RSV-infected SBPHs; small and large, the LsVg small or large subunit. Ab47Km and Ab111Km, the LsVg subunit-specific antibodies.
(TIF)

S2 Fig RSV or *E*. *coli* survival in the hemolymph of *L*. *striatellus*. *E*. *coli* or RSV was delivered into the *L*. *striatellus* hemolymph, and the phagocytosis ratio was calculated as the ratio of infected hemocytes to total hemocytes.
(TIF)

S3 Fig Western blots to determine the molecular weights and subunit distribution of proteins in the fat body (FB) or hemolymph (HL) of *L*. *striatellus*. Same amounts of the total proteins were fractionated by SDS-PAGE (10%) and probed with the subunit-specific antibodies Ab42K or Ab67K2. M, the molecular weight marker (kDa). Lines on the right, identified LsVg subunits.
(TIF)
