# Supplementary material for: Insect tissue-specific vitellogenin facilitates transmission of plant virus
Source: PLoS Pathog. 2018 Feb 23;14(2):e1006909. doi: 10.1371/journal.ppat.1006909 (PMC5849359; doi:10.1371/journal.ppat.1006909)
Supplement: S1 Table — (PDF) [file ppat.1006909.s001.pdf]

Table S1. Primers used in this study

Primers	Sequences (5'-3')	Purpose
LsVg-QF<br>LsVg-QR	TGGGTATGCACATGAAAGC<br>CCTAGACTTGATGGCGTTCT	*q*-PCR primers for *L.striatellus* vitellogenin
ef2-QF<br>ef2-QR	GTCTCCACGGATGGGCTTT<br>ATCTTGAATTTCTCGGCATACATTT	*q*-PCR primers for *L.striatellus* elongation factor 2
pc3-QF<br>pc3-QR	GATGCGTTGTCTTACCTGACTGC<br>CACTATCCCATACCTCGACACCA	*q*-PCR primers for RSV capsid protein pc3
47K-QF<br>47K-QR	TGGGTATGCACATGAAAGC<br>CCTAGACTTGATGGCGTTCT	*q*-PCR primers for LsVn small subunit
67K-QF<br>67K-QR	CACTCACCTCCTTCACTATCCTCA<br>CTTCTCATTCCTCACATCACCCT	*q*-PCR primers for LsVn medium subunit
111K-QF<br>111K-QR	GATGGTGAGACCCTTGTTGAGAT<br>ATGCACTTCTTGGGAGTGGTAAA	*q*-PCR primers for LsVn large subunit
VgN-si-F<br>VgN-si-R	TAATACGACTCACTATAGGTGTGAGACCCACTACGATGTT<br>TAATACGACTCACTATAGGCTGGAAGAGGAAGAGGAGGAG	Amplify N-terminus of *LsVg* for RNA silence
VgC-si-F<br>VgC-si-R	TAATACGACTCACTATAGGATGAAAGTAGCATTCCCCACC<br>TAATACGACTCACTATAGGGCCTCAAATGCGAATCCAGT	Amplify C-terminus of *LsVg* for RNA silence
gfp-si-F<br>gfp-si-R	TAATACGACTCACTATAGGATGGTAGATCTGACTAGTAA<br>TAATACGACTCACTATAGGCTAGTCATCTGCACCTTCTG	Amplify *GFP* for RNA silence
