# Supplementary figures and images for: Insect tissue-specific vitellogenin facilitates transmission of plant virus
Source: PLoS Pathog. 2018 Feb 23;14(2):e1006909. doi: 10.1371/journal.ppat.1006909 (PMC5849359; doi:10.1371/journal.ppat.1006909)

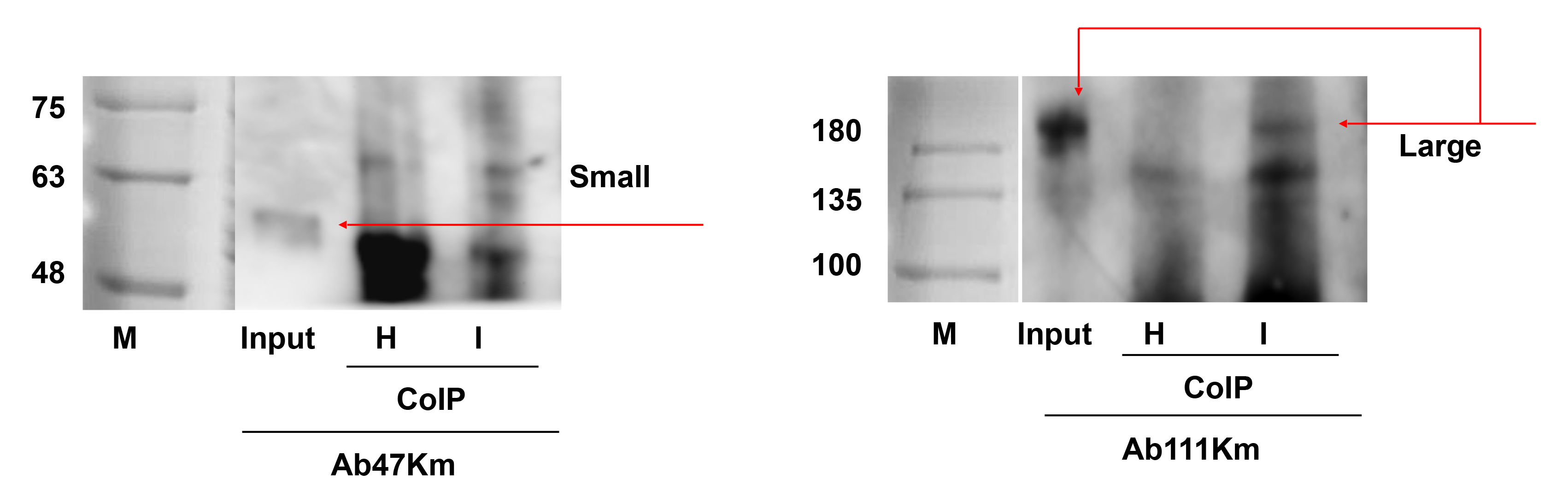

Supplement: S1 Fig — H, RSV-free SBPHs; I, RSV-infected SBPHs; small and large, the LsVg small or large subunit. Ab47Km and Ab111Km, the LsVg subunit-specific antibodies. (TIF) [file ppat.1006909.s002.tif]

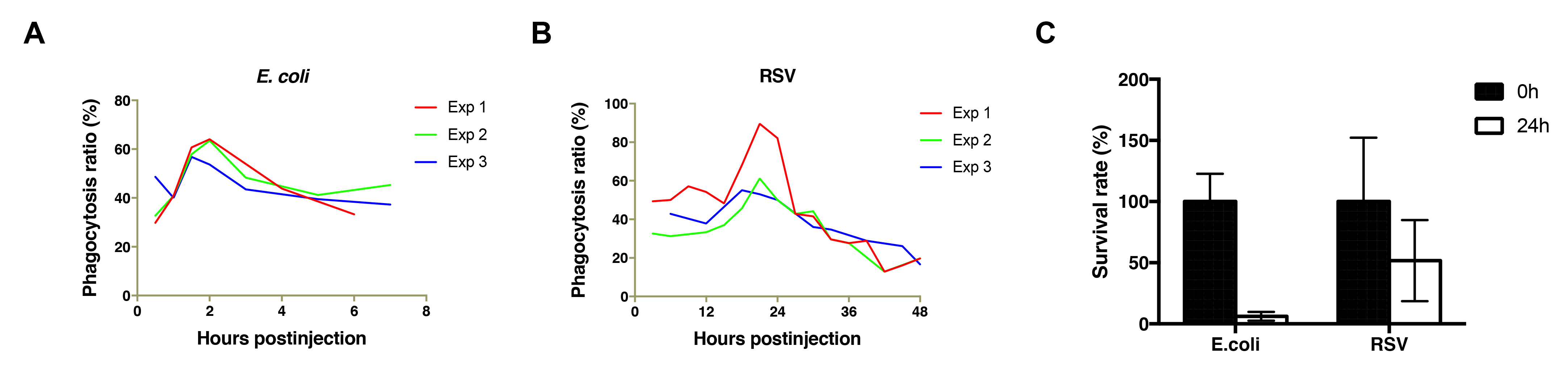

Supplement: S2 Fig — E. coli or RSV was delivered into the L. striatellus hemolymph, and the phagocytosis ratio was calculated as the ratio of infected hemocytes to total hemocytes. (TIF) [file ppat.1006909.s003.tif]

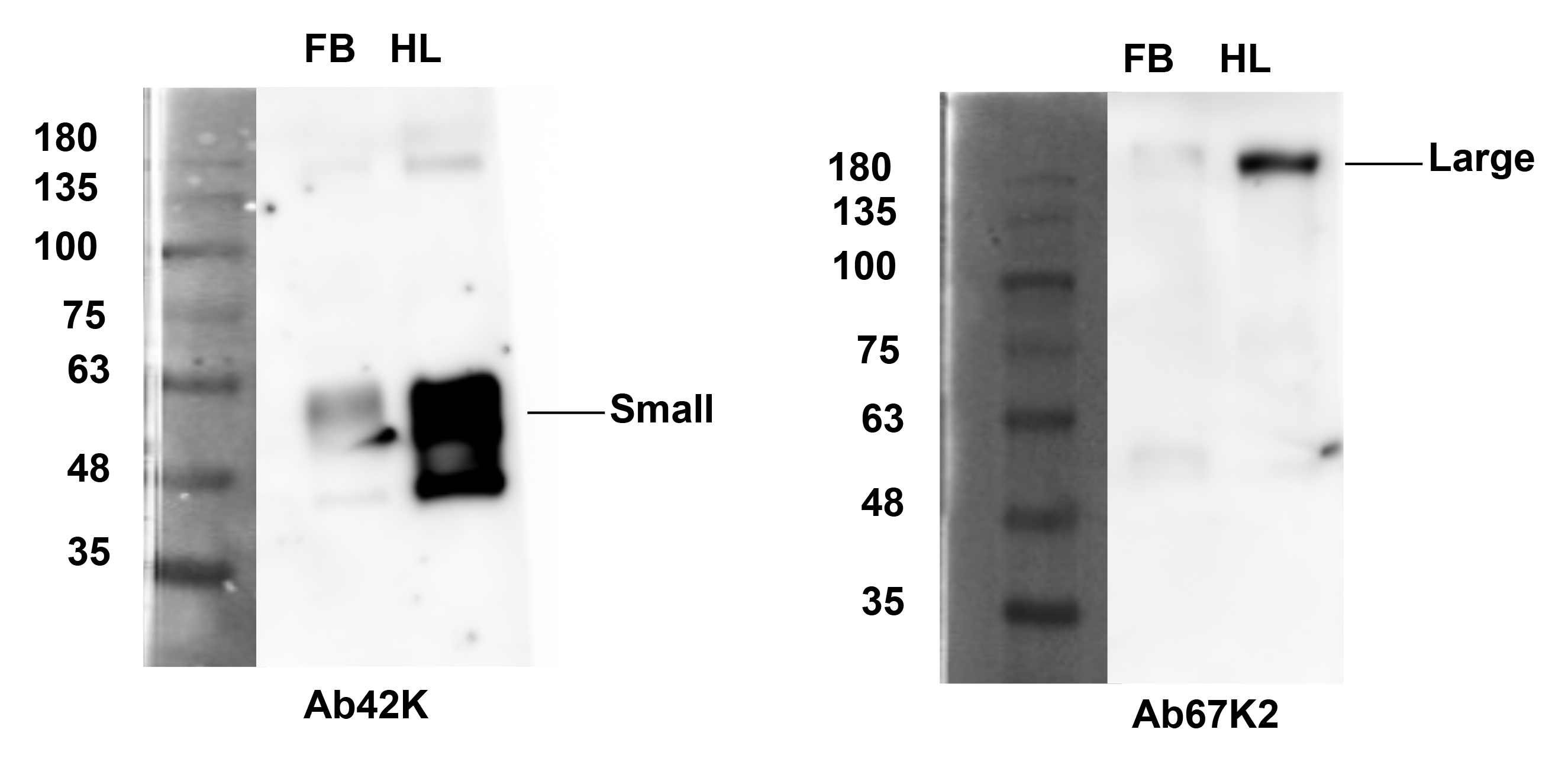

Supplement: S3 Fig — Same amounts of the total proteins were fractionated by SDS-PAGE (10%) and probed with the subunit-specific antibodies Ab42K or Ab67K2. M, the molecular weight marker (kDa). Lines on the right, identified LsVg subunits. (TIF) [file ppat.1006909.s004.tif]
